# The relationship between apoptosis, chromatin configuration, histone modification and competence of oocytes: A study using the mouse ovary-holding stress model

**DOI:** 10.1038/srep28347

**Published:** 2016-06-20

**Authors:** Juan Lin, Fei Chen, Ming-Ju Sun, Jiang Zhu, You-Wei Li, Liu-Zhu Pan, Jie Zhang, Jing-He Tan

**Affiliations:** 1 College of Animal Science and Veterinary Medicine, Shandong Agricultural University, Tai-an City 271018, P. R. China; 2 College of Life Science, Northeast Agricultural University, Harbin, 150030, P. R. China

## Abstract

The epigenetic factors causing competence differences between SN (surrounded nucleolus) and NSN (non-surrounded nucleolus) oocytes, the significance for the increased histone acetylation and methylation in SN oocytes, and whether chromatin configuration or histone modification determines oocyte competence, are unclear. This study has addressed these issues by using the ovary-holding (OH) stress models where oocyte SN configuration was uncoupled from histone modifications and developmental potential. Prepubertal mouse ovaries containing high percentages of NSN oocytes were preserved at 37 or 39 °C for 1 or 2 h before examination for oocyte chromatin configuration, developmental competence, histone modification and apoptosis. Whereas 1-h OH at 37 °C caused a moderate apoptosis with increased oocyte competence, improved histone modification and a normal NSN-to-SN transition, harsher OH conditions induced a severe apoptosis with decreased oocyte competence, impaired histone modification and a pseudo (premature) NSN-to-SN transition. Observations on Fas/FasL expression and using the gld (generalized lymphoproliferative disorder) mice harboring FasL mutations indicated that OH triggered oocyte apoptosis with activation of the Fas signaling. It was concluded that OH stress caused oocyte apoptosis with activation of the Fas/FasL system and that oocyte competence was more closely correlated with histone modification than with chromatin configuration.

With the growth of germinal vesicle (GV) stage mouse oocytes, their chromatin configuration changes from the non-surrounded nucleolus (NSN) pattern to the surrounded nucleolus (SN) pattern. While the NSN oocytes have diffuse chromatin, which does not form a heterochromatin rim around the nucleolus, the SN oocytes show condensed chromatin that is particularly confined around the nucleolus^1^,^2^,^3^,^4^. It seems that oocyte competence is influenced primarily by epigenetic factors that control overall gene expression and significantly modify GV chromatin configuration^5^,^6^,^7^,^8^. For example, fully-grown oocytes must end an NSN configuration before gaining full meiotic competence, and they must take on an SN configuration and stop gene transcription before being capable of blastocyst formation^6^. However, the mechanisms for the NSN-to-SN transition are largely unknown and the epigenetic factors causing the difference in developmental competence between NSN and SN oocytes remain to be clearly specified^9^.

Generally, histone acetylation leads to chromatin relaxation and thus correlates with gene activation, whereas histone deacetylation leads to chromatin condensation and therefore correlates with gene repression^10^. According to this rule, the core histone tails of GV chromatin should be less acetylated in SN than in NSN oocytes, because chromatin condenses and gene expression is silenced during oocyte growth. However, the level of histone acetylation in SN mouse oocytes was higher than that in NSN oocytes^11^,^12^,^13^. Furthermore, an increase in dimethylated H3K4 and H3K9 has been observed during the growth of mouse oocytes^12^, but its relationship with the NSN-to-SN transition of chromatin configuration and oocyte competence is unclear. Thus, the significance for the oocyte growth-associated increase in histone acetylation and methylation and its relationship with oocyte chromatin configuration and competence are unknown. Because it is impossible to observe such relations during normal oocyte growth/maturation, models where the SN configuration can be uncoupled from histone modification and oocyte competence must be used for such studies.

Our recent study showed that restraint of mice impaired the NSN-to-SN transition, histone acetylation and methylation in SN oocytes and oocyte developmental potential^14^. However, whereas the SN percentage of stressed oocytes returned to normal, neither the level of histone acetylation and methylation in SN oocytes nor the developmental competence recovered following a post-restraint recovery. Although these results suggested that the SN configuration was uncoupled from increased histone acetylation and methylation in the restraint-stressed oocytes, and that the developmental potential of SN oocytes is more closely correlated with epigenetic histone modification than with chromatin configuration, the conclusions need further verification using other models. Ovary holding (OH) either below body temperature^15^ or at a high temperature^16^ tended to increase SN configurations of oocytes. However, while the former induced early atretic changes and gave rise to more competent oocytes^17^, the latter totally abolished oocyte competence of maturation *in vitro*^16^. Thus, OH stress might be used to study the relationship between chromatin configuration, histone modification and competence of the SN oocytes, because the SN configuration is uncoupled from their developmental competence in the heat-stressed oocytes. Furthermore, why restraint stress inhibited while OH stress facilitated the NSN-to-SN transition, and how the early atretic changes triggered by the low temperature OH and other stressors^18^,^19^ improve oocyte competence, need further investigations.

Although observations suggest that the SN configuration represents a stage of GV oocyte that is more advanced toward ovulation, the possibility that this configuration could represent a step toward atresia has also been indicated in some reports^4^,^6^. We thus suspected that OH at a high temperature might have induced oocyte apoptosis with a pseudo NSN-to-SN transition reminiscent of the nuclear pyknosis in apoptotic somatic cells^20^,^21^, and the resulting configuration with condensed chromatin might have been mistaken as a “SN” configuration in previous reports^16^. On the other hand, the early atretic changes induced by the low temperature OH and other stressors might have improved oocyte competence by promoting a normal NSN-to-SN transition and improving histone modification.

Fas-mediated apoptosis plays a major role in the induction of apoptosis^22^,^23^,^24^. One recent study indicated that cumulus cells surrounding the aging oocyte released sFasL, which facilitated oocyte aging by binding to Fas receptors on the oocyte^25^. Furthermore, activation of the Fas pathway during *in vitro* maturation increased the incidence of apoptosis in cumulus cells of bovine oocytes^26^. Thus, it is worth studying whether the Fas/FasL system plays any role in the OH stress-induced apoptosis of ovarian oocytes.

We thus hypothesized that OH stress would trigger apoptosis of ovarian cells and oocytes by activating the Fas/FasL system. Whereas a moderate apoptosis induced by milder stresses would improve oocyte competence by facilitating a normal NSN-to-SN transition and improving histone modification, severe apoptosis induced by harsher stresses would decrease competence by inducing a pseudo (premature) NSN-to-SN transition and by impairing histone modification. The aim of this study was to test this hypothesis by OH of prepubertal mouse ovaries from both wild-type mice and the gld (generalized lymphoproliferative disorder) mice harboring FasL mutations. The prepubertal mouse ovaries without equine chorionic gonadotropin (eCG) priming were used because previous studies have shown that whereas adult mouse ovaries contain few, the prepubertal mouse ovaries contain many NSN oocytes before eCG treatment^27^. The FasL in gld mice carries a point mutation in the C-terminal region, and the recombinant gld FasL can not induce apoptosis in cells expressing Fas^28^. This suggests that the gld mice can be used as models to study roles of the Fas signaling in stress-induced apoptosis of ovarian cells.

## Results

### Effects of OH stress on GV chromatin configurations of mouse oocytes

**Figure 1:**
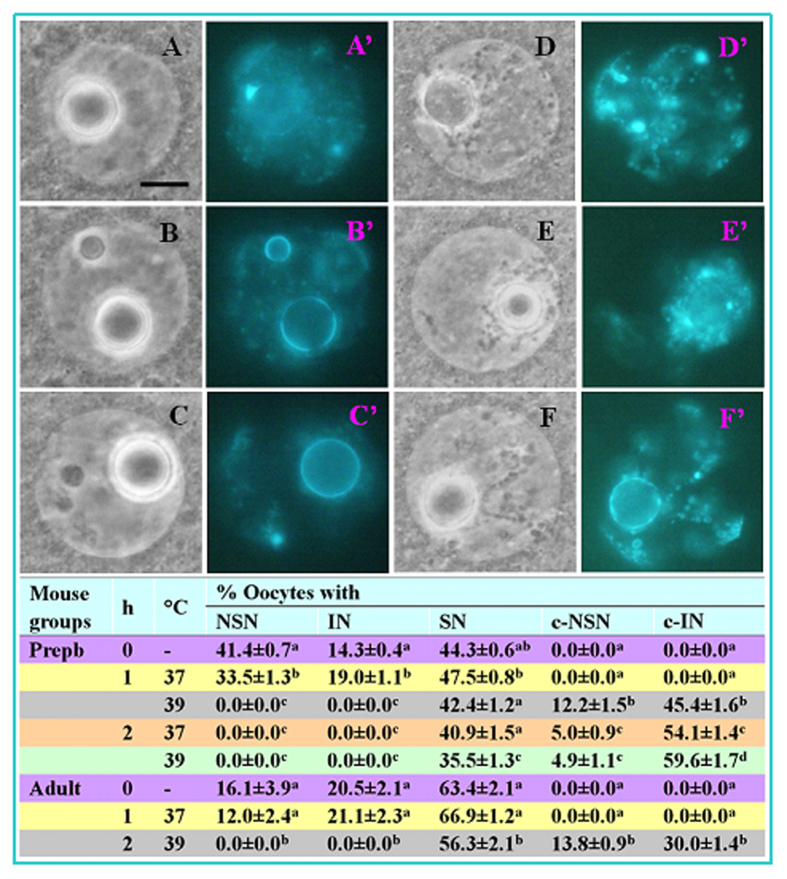
GV chromatin configurations of mouse oocytes before (0 h) or following OH for 1 or 2 h at 37 or 39 °C. Photographs (**A**) and A’, (**B**) and B’, (**C**) and C’, (**D**) and D’, (**E**) and E’, and (**F)** and F’ are the same oocytes observed with phase contrast and fluorescence, respectively, after Hoechst 33342 staining. A’, B’ and C’ show, respectively, oocytes with NSN, intermediate (IN) and SN configurations before OH. D’ shows an oocyte with condensed NSN (c-NSN) configuration, and E’ and F’ show oocytes with condensed IN (c-IN) configuration. Original magnification ×1300. Scale bar is 10 μm. The table shows percentages of oocytes with different chromatin configurations. a-d: Values without a common letter in their superscripts differ significantly (P < 0.05) in the same column. Each treatment was repeated 5 times with each replicate containing 50–60 oocytes.

Oocytes from newly recovered ovaries before OH showed 3 patterns of chromatin configuration. In the NSN pattern, chromatin was the least condensed, with sparse fine heterochromatin granules that did not surround the nucleolus (Fig. 1A’). In both the SN and the intermediate (IN) oocytes, nucleoli were enclosed by heterochromatin. However, while the chromatin in SN oocytes was the most condensed, with almost no diffuse chromatin (Fig. 1C’), the chromatin in IN oocytes was less condensed, always with some diffuse chromatin in the nucleoplasm (Fig. 1B’). After OH of prepubertal ovaries at 37 °C for 2 h or at 39 °C for 1 or 2 h, the diffuse chromatin in the NSN and IN oocytes condensed into big masses forming the condensed NSN (c-NSN, Fig. 1D’) and condensed IN (c-IN, Fig. 1E’,F’) configurations, respectively (Fig. 1, Table). Some of the NSN oocytes became either IN or SN oocytes after OH at 37 °C for 1 h. Similar changes in the chromatin configuration were observed in oocytes after OH of adult mouse ovaries, although the proportion of NSN oocytes was very low in the adult ovaries. Our live imaging further confirmed the NSN to c-NSN and IN to c-IN transition (Supplementary Fig. S1). The results suggested that although OH at 37 °C for 1 h facilitated a normal NSN-to-SN transition, OH at 37 °C for 2 h or at 39 °C for 1 or 2 h caused a pseudo (premature) NSN-to-SN transition, generating many oocytes with c-IN configurations.

### Effects of OH stress on maturation, activation and embryo development of mouse oocytes

When oocytes were recovered from fresh ovaries before OH, although none of the oocytes from prepubertal mice formed blastocysts, 40% of those from adult mice did (Table 1), confirming that the culture system used in this study could support maturation of mouse oocytes from ovaries that had not been stimulated with eCG. Compared to those in control oocytes recovered before OH, rates of oocyte maturation, activation and 4-cell embryos decreased significantly after OH at 39 °C for 1 or 2 h or at 37 °C for 2 h. After OH at 37 °C for 1 h, however, percentages of 4-cell embryos increased significantly compared to those in control oocytes, and 10% of the 4-cell embryos developed into blastocysts. The results suggested that although OH for a longer time or at a higher temperature impaired oocyte competence, a short-time OH at body temperature improved oocyte developmental potential.

### Effects of OH stress on GV chromatin histone modifications of mouse oocytes

**Figure 2:**
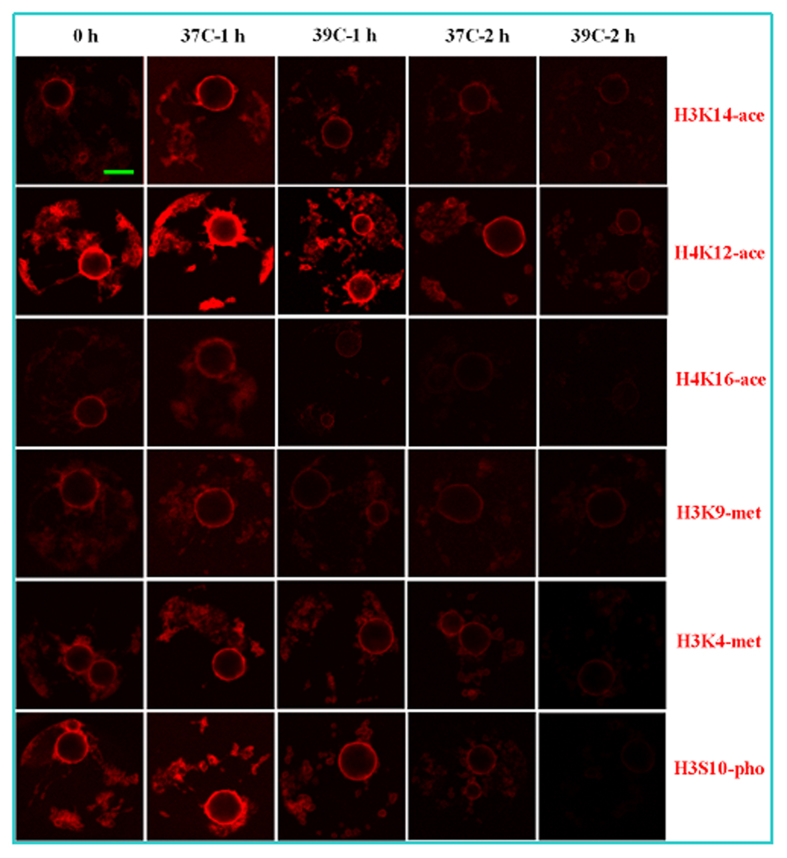
Laser confocal photomicrographs show acetylation (ace) of histone H3K14, H4K12 and H4K16, dimethylation (met) of H3K9 and H3K4, and phosphorylation (pho) of H3S10 in the SN oocytes before or after OH of prepubertal mouse ovaries at different temperatures for different times. Acetylated, dimethylated or phosphorylated histone was pseudo-colored red. Original magnification ×200. Scale bar is 10 μm.

In all the histone sites observed, levels of acetylation, dimethylation and phosphorylation increased in the NSN, IN and even SN oocytes after OH at 37 °C for 1 h, but they decreased in SN, c-NSN and c-IN oocytes after OH at 37 °C for 2 h or at 39 °C for 1 or 2 h (Table 2, Fig. 2). In the same histone site, the level of histone modification in the c-NSN and c-IN oocytes tended to be lower than that in the NSN and IN oocytes, respectively, indicating that the pseudo NSN-to-SN transition was associated with impaired histone modification. The acetylation level of H4K16 was apparently lower than that of H3K14 and H4K12 in oocytes with various chromatin configurations (Fig. 2). Together with our results shown in Table 1, it is suggested that in all the histone sites examined, the levels of histone modification were positively correlated with the developmental potential of oocytes after different OH treatments.

### Effects of OH stress on apoptosis of mural granulosa cells (MGCs), cumulus cells and oocytes

**Figure 3:**
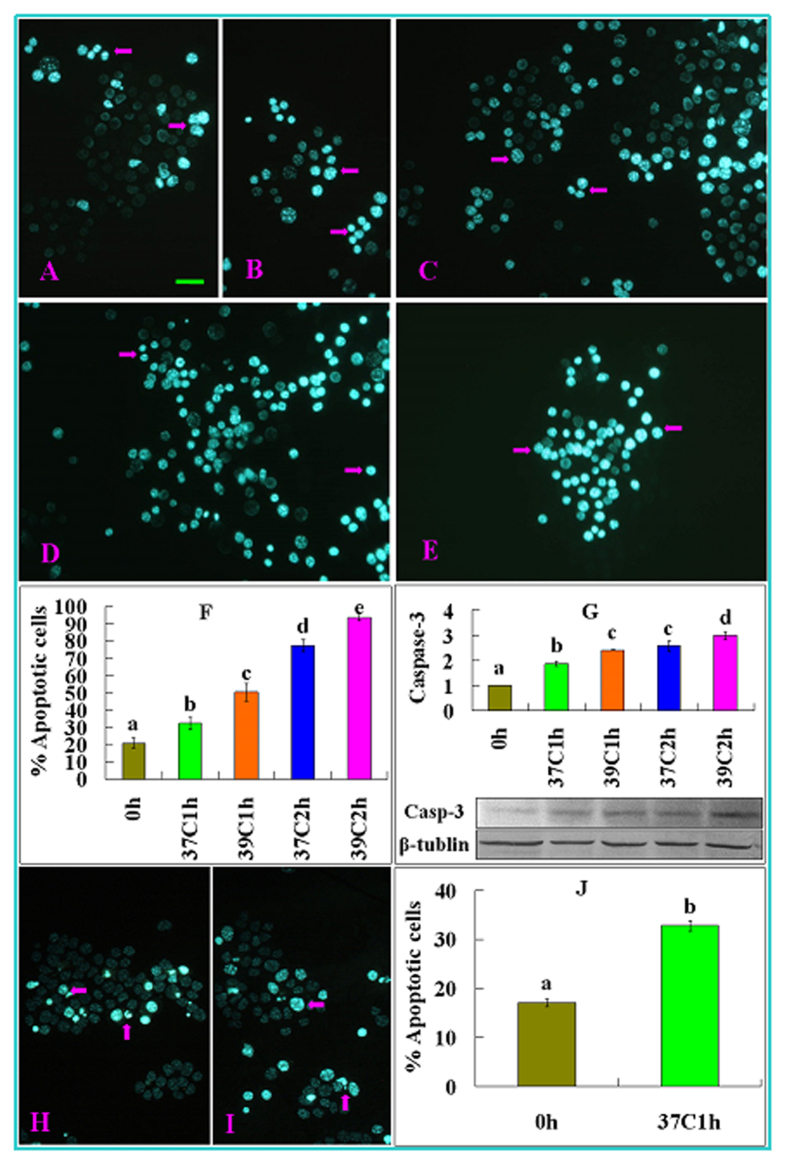
Apoptosis of MGCs and cumulus cells following OH of prepubertal mouse ovaries at different temperatures for different times. Micrographs (**A**–**E**) show MGCs smears and micrographs (**H**,**I**) show cumulus cells smears stained with Hoechst 33342 and observed under a fluorescence microscope. The heterochromatin was heavily stained with Hoechst and gave bright fluorescence. Whereas the apoptotic cells showed pyknotic nuclei full of heterochromatin (Arrows), healthy cells showed normal nuclei with sparse heterochromatin spots. Smears (**A**–**E**) show MGCs from ovaries before (**A**) or after OH at 37 °C for 1 h (**B**), 39 °C for 1 h (**C**), 37 °C for 2 h (**D**) and 39 °C for 2 h (**E**), respectively, while smears (**H**,**I**) show cumulus cells from ovaries before (**H**) and after OH at 37 °C for 1 h (**I**), respectively. Original magnification ×400. Scale bar is 20 μm. Graphs (**F**,**G**) show percentages of apoptotic cells and the level of active caspase-3 (Casp-3) in MGCs, respectively, and graph (**J**) shows percentages of apoptotic cumulus cells. In graph (**G**), the molecular weights of active caspase-3 and β-tubulin are 17 and 55 kDa, respectively. To measure percentages of apoptotic cells, each treatment was repeated 3–5 times with each replicate containing 10 smears, and to quantify the level of active caspase-3 by western blotting, each treatment was repeated 3 times with each replicate containing MGCs from 4 ovaries. a–e: Values without a common letter above their bars differ significantly (P < 0.05).

**Figure 4:**
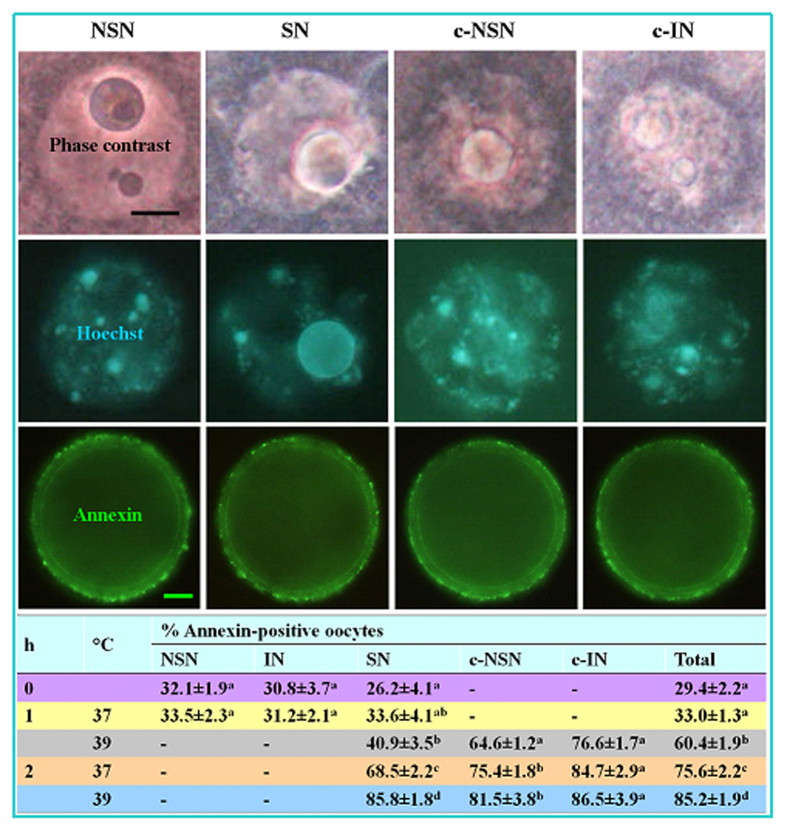
Annexin-positive oocytes with different chromatin configurations after OH of prepubertal mouse ovaries at different temperatures for different times. Photographs in the same column show the same oocytes observed under phase contrast (original magnification ×1300, scale bar is 10 μm) and fluorescence after Hoechst (×1300) and annexin (×400, scale bar is 15 μm) staining, respectively. Photographs in different columns show oocytes with NSN, SN, c-NSN and c-IN configurations, respectively. The table shows percentages of annexin-positive oocytes with various chromatin configurations after OH at 37 or 39 °C for 1 or 2 h. a–d: Values without a common letter in their superscripts differ (P < 0.05) in the same column. Each treatment was repeated 4-5 times with each replicate containing about 30 oocytes.

Both apoptotic percentages and active caspase-3 levels of MGCs were low before OH, but increased significantly with increasing harshness of OH conditions (Fig. 3). Percentages of annexin-positive (apoptotic) oocytes did not differ before OH and after OH at 37 °C for 1 h, but increased significantly with increasing harshness of OH conditions (Fig. 4). Furthermore, our Hoechst staining showed that the percentage of apoptotic cumulus cells increased significantly after OH at 37 °C for 1 h compared to that observed before OH (Fig. 3). Taken together, the results suggested that whereas a mild stress induced moderate apoptosis, severe stresses caused heavy apoptosis in mouse oocytes.

### Effects of OH stress on the expression of FasL in MGCs and Fas receptors in oocytes and cumulus cells

**Figure 5:**
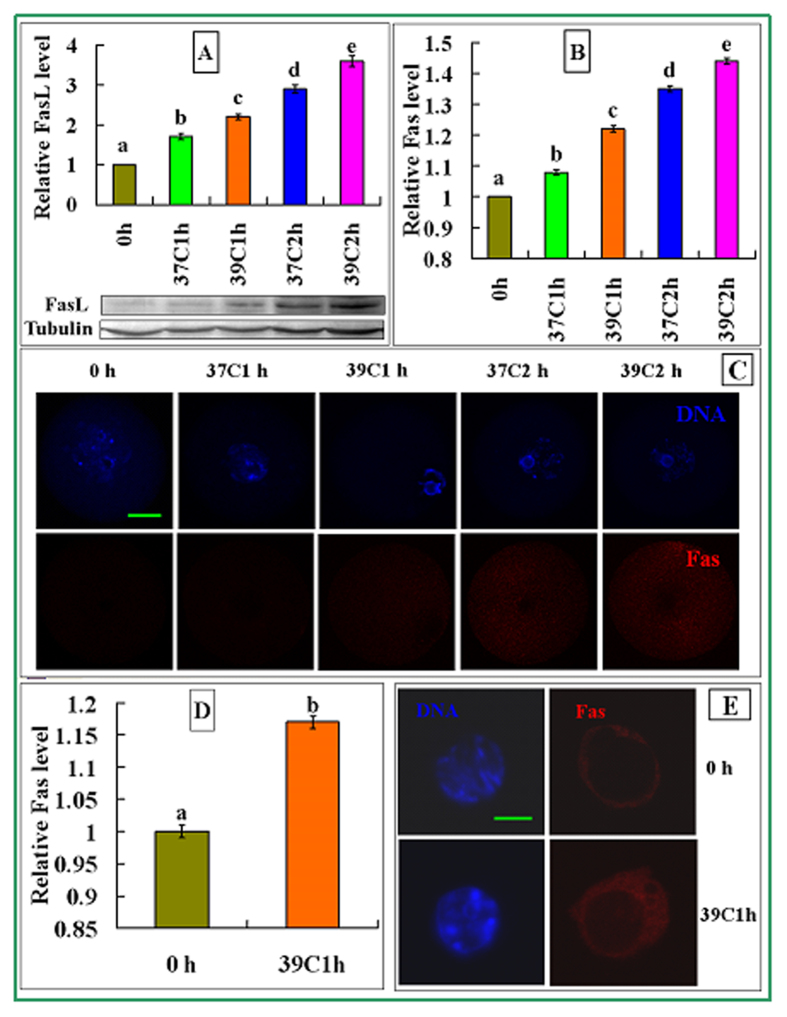
Levels of FasL in MGCs and Fas receptors in oocytes and cumulus cells after OH of prepubertal mouse ovaries. The OH conditions include before OH (0 h) and after OH at 37 °C for 1 h (37C1h), 39 °C for 1 h (39C1h), 37 °C for 2 h (37C2h) and 39 °C for 2 h (39C2h). Panel A shows FasL expression in MGCs revealed by western blotting. The molecular weights of FasL and β-tubulin (Tublin) are 31 and 55 kDa, respectively. Panel B shows Fas quantification by immunocytochemistry in oocytes. Each treatment was repeated 3–4 times with each replicate containing about 30 oocytes. Panel C shows confocal micrographs showing Fas localization in oocytes. Original magnification ×400. Scale bar is 30 μm. Panel D shows Fas quantification in cumulus cells by immunocytochemistry. Each treatment was repeated 3–4 times with each replicate containing about 30 cells from 15 images. Panel E shows confocal micrographs showing Fas localization in cumulus cells. Original magnification ×650. Scale bar is 5 μm. In the confocal images, DNA and Fas were pseudo colored blue and red, respectively. In the graphs, a–e indicates values without a common letter above their bars differ significantly (P < 0.05).

Both FasL in MGCs (Fig. 5A) and Fas receptors in oocytes (Fig. 5B) increased significantly with increasing harshness of OH conditions from before OH to OH at 37 °C for 1 h to 39 °C for 1 h to 37 °C for 2 h to 39 °C for 2 h. Fas receptors in cumulus cells also increased from before OH to OH at 39 °C for 1 h (Fig. 5D,E). The results suggested that the OH stress induced oocyte apoptosis by activating the Fas system in the ovary.

### Effects of OH stress on apoptosis and histone acetylation of oocytes from gld mice

**Figure 6:**
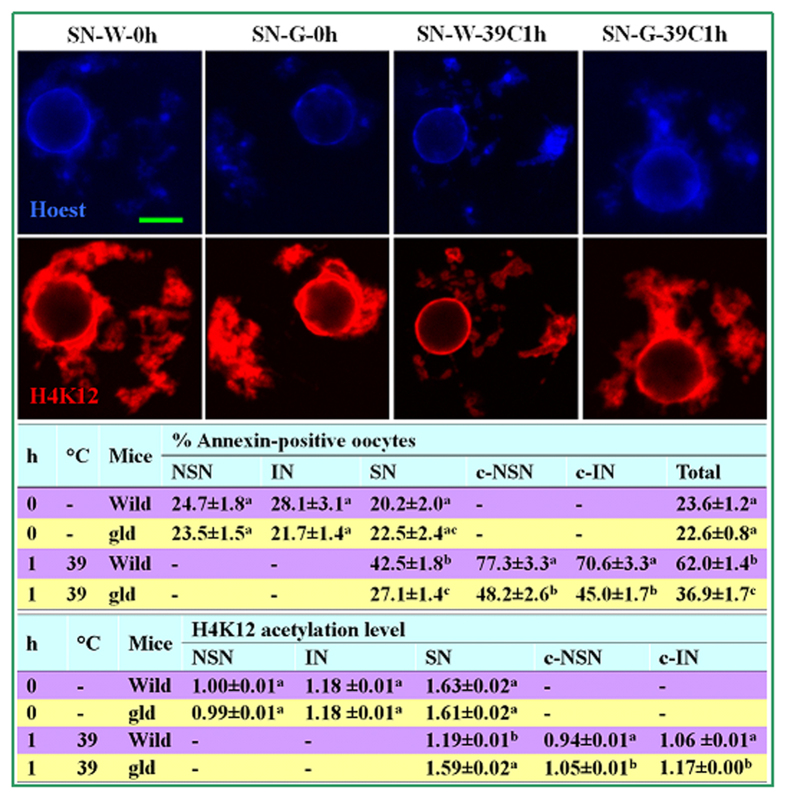
Percentages of annexin-positive oocytes and levels of H4K12 acetylation in oocytes with different chromatin configurations after OH under different conditions in wild-type and gld prepubertal mice. The micrographs are laser confocal images of SN oocytes in which Hoechst 33342 (upper row) and H4K12 (lower row) were pseudo-colored blue and red, respectively. Original magnification × 200. Scale bar is 10 μm. Images in the same column are from the same oocyte observed at different optical wavelengths. The SN oocytes shown in different columns include oocytes before OH (0 h) or after OH at 39 °C for 1 h (39C1h) from wild-type (W) or gld (G) mice. The two tables show % annexin-positive oocytes and relative levels of H4K12 acetylation among different oocytes, respectively. Each treatment was repeated 4 times with each replicate containing about 30 oocytes. a–c: Values in the same column without a common letter in their superscripts differ (P < 0.05).

Annexin-positive percentages and H4K12 acetylation levels in oocytes from freshly collected ovaries (0 h) did not differ between wild-type and gld mice (Fig. 6). Following OH at 39 °C for 1 h, however, whereas the annexin-positive percentages were significantly lower, levels of H4K12 acetylation were significantly higher in gld than in wild-type mice. The results further confirmed that OH stress triggered oocyte apoptosis and impaired epigenetic histone modification by activating the Fas system.

## Discussion

The present results showed that whereas a 1-h OH at body temperature promoted a normal NSN-to-SN transition, OH for a longer time or at a higher temperature caused a pseudo (premature) NSN-to-SN transition, generating the c-IN and c-NSN configurations. The c-IN and c-NSN configurations generated by the pseudo NSN-to-SN transition showed abnormally condensed chromatin masses, which were reminiscent of the pyknotic nuclei observed in apoptotic somatic cells. It is well known that apoptotic cell death in somatic cells is characterized by nuclear pyknosis (chromatin condensation) and DNA fragmentation, among other morphological features^20^,^21^. Whereas GV oocytes from young mice with a normal fertility had well-defined NSN and SN configurations, those from old mice with a reduced fertility had chromatin configurations that could not be classified as either NSN or SN^29^. From the images provided in that report, similarities are obvious between our c-IN and c-NSN configurations and their configurations that could not be classed as either NSN or SN. Furthermore, abnormal chromatin condensation has been observed following chemical treatment of oocytes. For example, when porcine oocytes were treated with roscovitine, the dispersed chromatin disappeared or condensed and the rim of the nucleolus became morphologically incomplete^30^.

In the present study, whereas OH at 37 °C for 1 h enhanced histone modification, OH at 37 °C for 2 h or at 39 °C for 1 or 2 h impaired histone modification in c-NSN, c-IN, and even in the SN oocytes. Our previous study demonstrated that restraint stress of mice decreased the levels of H3K14 and H4K12 acetylation and of H3K4 and H3K9 dimethylation in SN oocytes. Similar changes in histone acetylation and methylation were observed in oocytes from aged mice. For example, acetylation of H4K12 and H4K16 decreased in GV oocytes from aged mice compared to those in oocytes from young mice^31^. Whereas GV oocytes from young mice showed dimethylation of H3K4, H3K9, H3K36, H3K79 and H4K20, and trimethylation of H3K9, many GV oocytes from old mice lacked trimethylation of H3K9 and dimethylation of H3K36, H3K79 and H4K20^29^. Furthermore, feeding mice with mycotoxin decreased oocyte levels of H3K27 trimethylation and H4K20 dimethylation^32^, and exposure to bisphenol A during follicle culture significantly impaired H3K9 trimethylation^33^.

Our previous study showed that restraint stress of mice inhibited GV chromatin condensation and hindered the NSN-to-SN transition while increasing the level of H3S10 phosphorylation^14^. This is different from the present results that OH stress facilitated chromatin condensation and evoked a pseudo NSN-to-SN transition while decreasing the level of H3S10 phosphorylation. It is known that H3S10 phosphorylation plays a dual role during the cell cycle; whereas it maintains relaxed chromatin for active transcription in interphase, it facilitates chromatin condensation during mitosis^34^. Furthermore, while chronic ethanol consumption increased H3S10 phosphorylation, binge ethanol administration reduced its level of phosphorylation^35^. Thus, our restraint stress of mice over a 48-h period, which corresponds to a chronic stress, would have hindered the NSN-to-SN transition by increasing the level of H3S10 phosphorylation, whereas OH for 1 or 2 h is an acute severer stress, which would have enhanced chromatin condensation by reducing H3S10 phosphorylation in the interphase.

This study demonstrated that whereas OH at 37 °C for 1 h improved oocyte developmental potential, OH at 37 °C for 2 h or at 39 °C for 1 or 2 h decreased oocyte developmental competence. Our further observation indicated that although 1-h OH at 37 °C caused only a moderate apoptosis in MGCs and cumulus cells and no apoptosis in oocytes, OH at 37 °C for 2 h or at 39 °C for 1 or 2 h induced heavy apoptosis in MGCs and early apoptosis in oocytes. There have been numerous reports that a moderate apoptosis in MGCs or cumulus cells is associated with improved developmental potential of oocytes^18^,^19^. Notably, morulae/blastocysts rates of goat oocytes^36^ and blastocyst hatching rates of bovine oocytes^37^ were significantly higher in early atretic follicles than in the non- or late atretic follicles. However, the mechanisms for the beneficial effect of the early follicle atresia on oocyte developmental competence are not fully understood. The present results showed that the improved development observed in oocytes with moderately apoptotic MGCs and cumulus cells was associated with accelerated normal NSN-to-SN transition and enhanced histone modification, whereas the impaired development in oocytes with heavy apoptosis was characterized by a premature chromatin condensation (pseudo NSN-to-SN transition) and impaired histone modification. This suggests that early follicle atresia might have improved oocyte competence by accelerating histone modification as well as by promoting a normal NSN-to-SN transition.

The present results showed that while FasL was expressed in MGCs, Fas was expressed in oocytes and cumulus cells, and that both FasL and Fas increased significantly with increasing harshness of the OH conditions. Our observation on percentages of annexin-positive oocytes and levels of H4K12 acetylation indicated that oocytes from gld mice were less harmed by OH than oocytes from wild-type mice. Taken together, the results suggested that OH stress triggered oocyte apoptosis and impaired epigenetic histone modification and oocyte competence by activating the Fas system. Reports on Fas/FasL expression in ovaries are few and those on Fas expression in healthy oocytes are in conflict. In mice, for example, although expression of both Fas and FasL was observed in granulosa cells of both healthy and atretic follicles, Fas was observed only in oocytes of atretic follicles^38^. In bovine, however, Fas was expressed in normal immature oocytes, whereas FasL was expressed only in cumulus cells^26^,^39^. Thus, the present results confirmed Fas expression in healthy mouse oocytes.

In summary, for the first time, the present results showed that OH stress triggered oocyte apoptosis with activation of the Fas/FasL system. Whereas a moderate apoptosis induced by a mild OH stress improved oocyte competence with promoted NSN-to-SN transition and histone modifications, the heavy apoptosis induced by a harsh OH stress impaired oocyte developmental potential with a premature chromatin condensation (pseudo NSN-to-SN transition) and impaired histone modifications. The c-NSN and c-IN configurations generated by the pseudo NSN-to-SN transition could have been mistaken as “SN” configurations in previous reports. Oocytes with the c-NSN and c-IN configurations showed impaired histone modification and reduced developmental potential. Together with our results that the developmental potential of SN oocytes diminished with decreasing levels of histone modifications as OH conditions became harsher, it is suggested that oocyte competence is more closely correlated with histone modification than with chromatin configuration. These data are important for our understanding of the mechanisms by which stress impairs oocyte developmental potential and how the epigenetic factors influence oocyte cytoplasmic maturation.

## Methods

The experimental procedures were approved by the Animal Care and Use Committee of the Shandong Agricultural University P. R. China (Permit number: SDAUA-2001-0510). The methods were carried out in accordance with the approved guidelines. Unless otherwise specified, all chemicals and reagents used in the present study were purchased from Sigma Chemical Co. (St. Louis, MO, USA).

### Mice, OH and oocyte recovery

Mice for most of the experiments in this study are the Kunming strain, which were bred in our laboratory. The gld mice with a germline mutation F273L in FasL in a C57BL/6 J genomic background and the wild-type C57BL/6 J mice were obtained from the Key Laboratory of Stem Cell Biology, Shanghai Institute for Biological Sciences, China. The mice were kept in a room under a 14 L:10D photoperiod, with lights-off at 20:00.

Female mice 18–19 days or 8–12 weeks after birth were sacrificed to collect ovaries without eCG-stimulation. The two ovaries recovered from one mouse were placed in a 1.5-ml microfuge tube containing 1 ml pre-warmed M2 medium and stored for 1 or 2 h in an incubator with a humidified atmosphere of air and temperature set at either 37 °C or 39 °C. At the end of OH, the large follicles on the ovary were ruptured in M2 medium to release oocytes. Only oocytes larger than 70 μm in diameter and with a homogenous cytoplasm were used.

### Observation of oocyte chromatin configurations

Oocytes were stripped of their cumulus cells by pipetting in M2 and stained for 5 min with 10 μg/ml Hoechst 33342 in M2. The stained oocytes were then mounted on a glass slide and observed for GV chromatin configurations under a Leica DMLB fluorescence microscope.

### Time-lapse imaging

Cumulus-free oocytes were stained in M2 medium containing 50 ng/ml Hoechst 33342 for 10 min. The oocytes were then cultured at 37 °C or 39 °C in HCZB medium containing 20 ng/ml Hoechst 33342 in an incubator (INUBG2E-ZILCS; Tokai Hit) and observed under a video microscope (DMI6000B; Leica). Time-lapse analysis was performed over a total of 3 h period. Phase contrast and fluorescence images of oocytes were taken at 15-min intervals with a charge-coupled device camera (885 EM; Andor), and the chromatin was pseudo-colored red.

### Oocyte maturation *in vitro*

Because mouse oocytes recovered without eCG priming mature poorly *in vitro*, oocyte maturation was conducted on cumulus cell monolayer in this study to improve maturation of such oocytes. Briefly, when cumulus cells grew to 80% of confluence, the spent medium in the wells of a 96-well culture plate was replaced with 100 μl maturation medium and then equilibrated for 3 h in a CO_2_ incubator. After being washed three times in M2 and once in the maturation medium, the oocytes were placed in the wells (around 35 each well) and culture at 37 °C in a humidified atmosphere of 5% CO_2_ in air. The maturation medium used was TCM-199 (Gibco, Grand Island, NY) supplemented with 10% (v/v) FBS (Gibco), 1 μg/ml 17β-estradiol, 24.2 mg/ml sodium pyruvate, 0.05 IU/ml FSH, 0.05 IU/ml LH and 10 ng/ml EGF.

### Oocyte activation and embryo culture

At 24 h of maturation culture, oocytes were stripped of cumulus cells by pipetting in M2 containing 0.1% hyaluronidase. After being washed twice in M2 and once in the activating medium (Ca^2+^ -free CZB medium supplemented with 10 mM SrCl_2_ and 5 μg/ml cytochalasin B), the oocytes were incubated in the activating medium for 6 h at 37 °C in a humidified atmosphere with 5% CO_2_ in air. At the end of activation treatment, the oocytes were examined with a microscope for the evidence of activation. Oocytes were considered activated when each contained one or two well-developed pronuclei. Activated oocytes were cultured for 4 days in regular CZB (30–35 oocytes per 100-μl drop) at 37 °C under humidified atmosphere with 5% CO_2_ in air. Glucose (5.5 mM) was added to CZB when embryos developed beyond 3- or 4-cell stages. Embryos were examined at 48 h and 96 h after activation treatment to record the numbers of 4-cell embryos and blastocysts, respectively.

### Immunofluorescence for detection of histone modifications and Fas expression

Procedures used for immunofluorescence were those reported in our previous study^14^. Briefly, oocytes were stripped of cumulus cells by pipetting in M2. The cumulus-free oocytes and cumulus cells were (1) fixed for 1 h with 4% paraformaldehyde; (2) permeabilized (oocytes only) with 0.5% (0.1% for Fas detection) Triton X-100 for 15 min (1 h for H3S10 phosphorylation); (3) blocked for 1 h in D-PBS containing 3% BSA and 0.1% Tween-20; (4) incubated at 4 °C overnight with primary antibodies; (5) incubated for 1 h with secondary antibodies; (6) stained for 5 min with 10 μg/ml Hoechst 33342 to visualize the DNA; and (7) mounted on a glass slide and observed with a laser confocal microscope (TCS SP2; Leica Microsystems). When mounting oocytes onto slides, care was taken to ensure that oocytes on different slides were compressed to the same extent and had a similar thickness. Blue diode (405 nm) and helium/neon (He/Ne; 543 nm) lasers were used to excite Hoechst and Cy^TM3^, respectively. Fluorescence was detected with the following bandpass emission filters: 420–480 nm (Hoechst) and 550–570 nm (Cy^TM3^), and the captured signals were recorded as blue and red, respectively.

All the oocytes used to observe modifications in the same histone site were processed and observed on the same day. The relative levels of histone acetylation, methylation and phosphorylation and Fas expression were quantified by measuring fluorescence intensities. For each experimental series, all the images were acquired with identical settings. Within the GV of each oocyte, a single plane with maximum amount of chromatin and maximum fluorescence intensity was selected to take photograph for further analysis. Relative intensities were measured on the raw images using the Image-Pro Plus software (Media Cybernetics Inc., Silver Spring, MD) under fixed thresholds across all the slides. Both the fluorescence density and the area of the objects giving the fluorescence were measured and the mean relative intensity of fluorescence was calculated for each oocyte. For each histone modification site, the average relative fluorescence of the NSN oocytes from freshly collected ovaries without holding was set to 1 and that of oocytes from other treatments were expressed relative to this value. The average Fas relative fluorescence of cumulus cells from unpreserved ovaries was set to 1, and those from preserved ovaries were expressed relative to this value.

The primary antibodies included Anti-acetyl-H3 (Lys14) (1:200 dilution, Millipore,06-911), Acetyl Histone H4K12 Polyclonal Antibody (1:1000 dilution, Epigentek, A-4029), Acetyl Histone H4K16 Polyclonal Antibody (1:50 dilution, Epigentek, A-4030), Histone H3K4 Dimethyl Polyclonal Antibody (1:200 dilution, Epigentek, A-4032), Histone H3K9 Dimethyl Polyclonal Antibody (1:100 dilution, Epigentek, A-4035), Anti-phospho-Histone H3 (Ser10) Antibody, clone RR002 (1:100 dilution, Millipore, 05-598), and rabbit polyclonal anti-Fas antibody (dilution 1:100 for oocytes and 1:75 for cumulus cells, Abcam, ab82419). The secondary antibodies used to detect acetylation in H3K14 and H4K12, methylation in H3K4 and H3K9 and Fas were Cy^TM3^-conjugated AffiniPure Goat Anti-Rabbit IgG (H + L) (1:800 dilution, Jackson ImmunoRerearsh, 111-165-144), that used to detect acetylation in H4K16 was Cy^TM3^-conjugated Donkey Anti-Goat IgG (H + L) (1:800 dilution, Jackson ImmunoRerearsh, 705-165-147), and that for detecting phosphorylation in H3S10 was Cy^TM3^-conjugated AffiniPure Goat Anti-Mouse IgG (H + L) (1:800 dilution, Jackson ImmunoRerearsh, 115-165-003).

### Annexin-V staining

The staining was performed using Annexin V-FITC Apoptosis Detection Kit (Biotool, B32115) according to the manufacturer’s instructions. During the whole process, reagents were kept at 37 °C to avoid false positives. Oocytes were placed in 100 μl 1 × binding buffer containing 5 μl Annexin-V/FITC and 5 μl propidium iodide, and incubated at room temperature for 15 min in the dark. After incubation, 400 μl of 1× binding buffer was added, and then the samples were mixed gently and kept on ice or directly mounted on siliconized slides with vaseline bridges and observed under a fluorescent microscope. After treatment with both probes, apoptotic oocytes showed green fluorescence, dead oocytes showed both green and red fluorescence, and healthy oocytes showed little or no fluorescence. Oocytes with more than 20% of the plasma membrane showing green fluorescence were considered annexin-positive. Dead oocytes were not taken into account. Immediately after observation, the oocytes were carefully released from the slides and processed for detection of histone modifications as described above.

### Apoptosis assessment in mural granulosa cells (MGCs) and cumulus cells

The MGCs clumps released into M2 medium at puncture of follicles were collected and pipetted with a thin pipette in M2 medium to separate individual cells. Cumulus cells were prepared by pipetting the cumulus-oocyte complexes with a thin pipette in M2. The MGCs and cumulus cells were then transferred to a 0.5-ml microfuge tube along with the M2 medium and centrifuged for 5 min at 200× g. After centrifugation, the MGCs pellets were resuspended in 50 μl of M2 medium with 10 μg/ml of Hoechst 33342 and stained in the dark for 5 min. The stained cells were then centrifuged for 5 min at 200× g. After removal of approximately half the supernatant, a 5-μl drop of suspension was smeared on a slide and observed under a Leica DMLB fluorescence microscope. Six to eight fields were randomly examined on each smear, and the percentages of apoptotic cells were calculated from more than 60 cells observed in each field. All images were reviewed by 2 investigators in a double blind manner.

### Western blot analysis

MGCs were placed in a 0.5-ml microfuge tube containing 20 μl sample buffer (20 mM Hepes, 100 mM KCl, 5 mM MgCl_2_, 2 mM DTT, 0.3 mM phenylmethyl sulfonyl fluoride, 3 μg/ml leupetin, 5 mM NaF, PH 7.5) and frozen at −80 °C until use. For protein extraction, 5 μl 5 × SDS-PAGE loading buffer was added to each tube, and the tubes were heated at 100 °C for 5 min. Total proteins were separated on a 12% (for FasL) or 15% (for active caspase-3) polyacrylamide gel by SDS-PAGE and transferred electrophoretically on to PVDF membranes. The membranes were then (1) washed twice in TBST (150 mM NaCl, 2 mM KCl, 25 mM Tris, 0.05% Tween 20, pH 7.4) and blocked for 1 h with TBST containing 3% BSA; (2) incubated at 4 °C overnight with rabbit polyclonal anti-Fas Ligand antibody (1:1000, ab15285, Abcam Co. Ltd.) or rabbit polyclonal anti-caspase-3 antibody (1:500, ab13847, Abcam Co. Ltd.) and mouse anti-β-tubulin monoclonal antibodies (1:1000, 05-661, Merck Millipore); (3) incubated for 1.5 h at 37 °C with alkaline phosphatase conjugated goat anti-rabbit IgG (1:1000, cw0111, Kangweishiji Biotechnology Co. Ltd., Beijing, China) and goat anti-mouse IgG (1:1000, cw0110, Kangweishiji Biotechnology Co. Ltd., Beijing, China) antibodies; (4) signals were detected by a BCIP/NBT alkaline phosphatase color development kit (Beyotime Institute of Biotechnology, Haimen City, China). The relative quantities of proteins were determined with Image J software by analyzing the sum density of each protein band image. The quantity of β-tubulin was used for internal control. The density value of each sample was normalized to its β-tubulin density value to get its relative quantity value. The relative quantity values of FasL or active caspase-3 in control ovaries before OH were set as 1 and the other values were expressed relative to this quantity. Each treatment was repeated 3 times with each replicate containing MGCs from 4 ovaries.

### Data analysis

There were at least three replicates for each treatment unless otherwise stated. Percentage data were arc sine transformed, and analyzed with ANOVA; a Duncan multiple comparison test was used to locate differences. The software used was Statistics Package for Social Sciences (SPSS 11.5, SPSS Inc. Chicago, IL). Data were expressed as mean ± SEM and P < 0.05 were considered significant.

## Additional Information

**How to cite this article**: Lin, J. *et al*. The relationship between apoptosis, chromatin configuration, histone modification and competence of oocytes: A study using the mouse ovary-holding stress model. *Sci. Rep.* **6**, 28347; doi: 10.1038/srep28347 (2016).

## Supplementary Material

Supplementary Information

## Figures and Tables

**Table 1:** Maturation, activation and embryo development *in vitro* of mouse oocytes after OH at different temperatures for different times.

Mouse groups	h	°C	% MII/cultured oocytes	% Activated/matured oocytes	% 4-cell embryos/activated oocytes	% Blastocysts/4-cell embryos
Prepb	0	–	87.0 ± 1.1^a^	72.6 ± 2.9^a^	42.5 ± 3.9^a^	0.0 ± 0.0^a^
	1	37	86.0 ± 1.3^a^	76.4 ± 1.9^a^	51.7 ± 3.1^b^	10.1 ± 1.2^b^
		39	66.8 ± 4.0^b^	49.7 ± 3.3^b^	14.1 ± 1.7^c^	0.0 ± 0.0^a^
	2	37	52.3 ± 3.1^c^	33.1 ± 1.3^c^	0.0 ± 0.0^d^	0.0 ± 0.0^a^
		39	0.0 ± 0.0^d^	0.0 ± 0.0^d^	0.0 ± 0.0^d^	0.0 ± 0.0^a^
Adult	0	–	89.6 ± 2.0^a^	82.2 ± 3.1^a^	54.5 ± 2.5^a^	38.1 ± 3.6^a^
	1	37	93.4 ± 1.7^a^	80.9 ± 0.4^a^	57.8 ± 1.8^a^	48.9 ± 2.4^b^
	2	39	0.0 ± 0.0^b^	0.0 ± 0.0^b^	0.0 ± 0.0^b^	0.0 ± 0.0^c^

^a–d^In the same column, values without a common letter in their superscripts differ significantly (P < 0.05) within mouse groups. Each treatment was repeated 5–6 times with each replicate including 35 oocytes.

**Table 2:** Relative levels of acetylation (ace), dimethylation (met) or phosphorylation (pho) in different histone sites in oocytes with various chromatin configurations after OH at different temperatures for different times*.

Histone site	h	°C	NSN	IN	SN	c-NSN	c-IN
H3K14-ace	0	–	1.00 ± 0.00^a^	1.07 ± 0.04^a^	1.33 ± 0.01^a^	–	–
	1	37	1.05 ± 0.01^b^	1.21 ± 0.02^b^	1.49 ± 0.02^b^	–	–
		39	–	–	1.23 ± 0.01^c^	1.00 ± 0.01^a^	1.05 ± 0.01^a^
	2	37	–	–	1.13 ± 0.02^d^	0.96 ± 0.01^a^	1.02 ± 0.01^b^
		39	–	–	1.07 ± 0.01^e^	0.88 ± 0.10^a^	1.01 ± 0.01^b^
H4K12-ace	0		1.00 ± 0.01^a^	1.12 ± 0.02^a^	1.49 ± 0.02^a^	–	–
	1	37	1.12 ± 0.02^b^	1.29 ± 0.01^b^	1.66 ± 0.02^b^	–	–
		39	–	–	1.27 ± 0.01^c^	1.00 ± 0.01^a^	1.08 ± 0.01^a^
	2	37	–	–	1.19 ± 0.01^d^	0.95 ± 0.01^b^	1.01 ± 0.01^b^
		39	–	–	1.03 ± 0.01^e^	0.94 ± 0.01^b^	0.97 ± 0.00^c^
H4K16-ace	0		1.00 ± 0.01^a^	1.06 ± 0.01^a^	1.28 ± 0.01^a^	–	–
	1	37	1.00 ± 0.02^a^	1.11 ± 0.01^b^	1.42 ± 0.01^b^	–	–
		39	–	–	1.13 ± 0.01^c^	0.99 ± 0.01^a^	1.03 ± 0.01^a^
	2	37	–	–	1.06 ± 0.01^d^	0.99 ± 0.01^a^	1.01 ± 0.01^b^
		39	–	–	1.03 ± 0.01^e^	0.97 ± 0.01^a^	0.93 ± 0.01^b^
H3K9-met	0	–	1.00 ± 0.01^a^	1.02 ± 0.01^a^	1.22 ± 0.01^a^	–	–
	1	37	1.01 ± 0.01^a^	1.22 ± 0.01^b^	1.31 ± 0.01^b^	–	–
		39	–	–	1.10 ± 0.01^c^	0.97 ± 0.01^a^	1.05 ± 0.01^a^
	2	37	–	–	1.05 ± 0.01^d^	0.94 ± 0.01^ab^	1.03 ± 0.01^b^
		39	–	–	0.98 ± 0.01^e^	0.92 ± 0.01^b^	0.97 ± 0.00^c^
H3K4-met	0	–	1.00 ± 0.00^a^	1.16 ± 0.01^a^	1.33 ± 0.01^a^	–	–
	1	37	1.08 ± 0.01^b^	1.19 ± 0.01^a^	1.41 ± 0.01^b^	–	–
		39	–	–	1.25 ± 0.01^c^	1.04 ± 0.01^a^	1.14 ± 0.01^a^
	2	37	–	–	1.19 ± 0.01^d^	0.97 ± 0.01^b^	1.12 ± 0.01^b^
		39	–	–	0.99 ± 0.01^e^	0.98 ± 0.02^b^	0.96 ± 0.01^c^
H3S10-pho	0	–	1.00 ± 0.01^a^	1.20 ± 0.02^a^	1.51 ± 0.02^a^	–	–
	1	37	1.11 ± 0.02^b^	1.33 ± 0.02^b^	1.89 ± 0.02^b^	–	–
		39	–	–	1.33 ± 0.02^c^	0.99 ± 0.01^a^	1.15 ± 0.02^a^
	2	37	–	–	1.16 ± 0.02^d^	0.95 ± 0.01^b^	1.05 ± 0.02^b^
		39	–	–	0.97 ± 0.01^e^	0.88 ± 0.01^c^	0.93 ± 0.01^c^

^*^Each treatment was repeated 3 times with each replicate containing 20–30 oocytes from prepubertal ovaries. For each histone site, the average relative fluorescence of the NSN oocytes before OH (0 h) was set to 1, and the averages of oocytes from other treatments within the same histone site were expressed relative to this value.

^a–e^Values without a common letter in their superscripts differ (P < 0.05) in the same column within histone sites.

## References

[b1] Mattson B. A. & Albertini D. F. Oogenesis: chromatin and microtubule dynamics during meiotic prophase. Mol Reprod Dev 25, 374–383 (1990). 1691651 10.1002/mrd.1080250411

[b2] Wickramasinghe D., Ebert K. M. & Albertini D. F. Meiotic competence acquisition is associated with the appearance of M-phase characteristics in growing mouse oocytes. Dev Biol 143, 162–172 (1991). 1985016 10.1016/0012-1606(91)90063-9

[b3] Debey P. . Competent mouse oocytes isolated from antral follicles exhibit different chromatin organization and follow different maturation dynamics. Mol Reprod Dev 36, 59–74 (1993). 8398131 10.1002/mrd.1080360110

[b4] Zuccotti M., Piccinelli A., Giorgi R. P., Garagna S. & Redi C. A. Chromatin organization during mouse oocyte growth. Mol Reprod Dev 41, 479–485 (1995). 7576615 10.1002/mrd.1080410410

[b5] De L. & Fuente R. Chromatin modifications in the germinal vesicle (GV) of mammalian oocytes. Dev Biol 292, 1–12 (2006). 16466710 10.1016/j.ydbio.2006.01.008

[b6] Tan J. H. . Chromatin configurations in the germinal vesicle of mammalian oocytes. Mol Hum Reprod 15, 1–9 (2009). 19019837 10.1093/molehr/gan069

[b7] De L., Fuente R., Baumann C. & Viveiros M. M. Chromatin structure and ATRX function in mouse oocytes. Results Probl Cell Differ 55, 45–68 (2012). 22918800 10.1007/978-3-642-30406-4_3

[b8] Bonnet-Garnier A. . Genome organization and epigenetic marks in mouse germinal vesicle oocytes. Int J Dev Biol 56, 877–887 (2012). 23417410 10.1387/ijdb.120149ab

[b9] Inoue A., Nakajima R., Nagata M. & Aoki F. Contribution of the oocyte nucleus and cytoplasm to the determination of meiotic and developmental competence in mice. Hum Reprod 23, 1377–1384 (2008). 18367455 10.1093/humrep/den096

[b10] Kallin E. & Zhang Y. Chromatin remodeling in: Lennarz W. J., Lane M. D. (eds), Encyclopedia of Biological Chemistry. Burlington, MA: Academic Press, 456–463 (2004).

[b11] De L. . Major chromatin remodeling in the germinal vesicle (GV) of mammalian oocytes is dispensable for global transcriptional silencing but required for centromeric heterochromatin function. Dev Biol 275, 447–458 (2004). 15501230 10.1016/j.ydbio.2004.08.028

[b12] Kageyama S. . Alterations in epigenetic modifications during oocyte growth in mice. Reproduction 133, 85–94 (2007). 17244735 10.1530/REP-06-0025

[b13] Ola S. I. . Meiotic competence and acetylation pattern of UV light classified mouse antral oocytes after meiotic arrest with isobutylmethylxanthine. Mol Reprod Dev 74, 591–599 (2007). 17034048 10.1002/mrd.20625

[b14] Wu X. F. . Restraint stress on female mice diminishes the developmental potential of oocytes: roles of chromatin configuration and histone modification in germinal vesicle stage oocytes. Biol Reprod 92, 13 (2015). 25411393 10.1095/biolreprod.114.124396

[b15] Pedersen H. G., Watson E. D. & Telfer E. E. Effect of ovary holding temperature and time on equine granulosa cell apoptosis, oocyte chromatin configuration and cumulus morphology. Theriogenology 62, 468–480 (2004). 15226003 10.1016/j.theriogenology.2003.10.006

[b16] Liu Y. . Germinal vesicle chromatin configurations of bovine oocytes. Microsc Res Tech 69, 799–807 (2006). 16886230 10.1002/jemt.20349

[b17] Blondin P., Coenen K., Guilbault L. A. & Sirard M. A. *In vitro* production of bovine embryos: developmental competence is acquired before maturation. Theriogenology 47, 1061–1075 (1997). 16728056 10.1016/s0093-691x(97)00063-0

[b18] Hagemann L. J. . Development during single IVP of bovine oocytes from dissected follicles: interactive effects of estrous cycle stage, follicle size and atresia. Mol Reprod Dev 53, 451–458 (1999). 10398421 10.1002/(SICI)1098-2795(199908)53:4<451::AID-MRD11>3.0.CO;2-3

[b19] de Wit A. A., Wurth Y. A. & Kruip T. A. Effect of ovarian phase and follicle quality on morphology and developmental capacity of the bovine cumulus-oocyte complex. J Anim Sci 78, 1277–1283 (2000). 10834583 10.2527/2000.7851277x

[b20] Reed J. C. Regulation of apoptosis by bcl-2 family proteins and its role in cancer and chemoresistance. Curr Opin Oncol 7, 541–546 (1995). 8547403 10.1097/00001622-199511000-00012

[b21] Ghavami S. . Autophagy and apoptosis dysfunction in neurodegenerative disorders. Prog Neurobiol 112, 24–49 (2014). 24211851 10.1016/j.pneurobio.2013.10.004

[b22] Dhein J., Walczak H., Baumler C., Debatin K. M. & Krammer P. H. Autocrine T-cell suicide mediated by APO-1/(Fas/CD95). Nature 373, 438–441 (1995). 7530335 10.1038/373438a0

[b23] Ju S. T. . Fas(CD95)/FasL interactions required for programmed cell death after T-cell activation. Nature 373, 444–448 (1995). 7530337 10.1038/373444a0

[b24] Poulaki V., Mitsiades C. S. & Mitsiades N. The role of Fas and FasL as mediators of anticancer chemotherapy. Drug Resist Updat 4, 233–242 (2001). 11991678 10.1054/drup.2001.0210

[b25] Zhu J. . Cumulus cells accelerate oocyte aging by releasing soluble Fas Ligand in mice. Sci Rep 5, 8683 (2015). 25731893 10.1038/srep08683 PMC4346792

[b26] Rubio Pomar F. J. . Role of Fas-mediated apoptosis and follicle-stimulating hormone on the developmental capacity of bovine cumulus oocyte complexes *in vitro*. Biol Reprod 71, 790–796 (2004). 15128594 10.1095/biolreprod.104.028613

[b27] Bouniol-Baly C. . Differential transcriptional activity associated with chromatin configuration in fully grown mouse germinal vesicle oocytes. Biol Reprod 60, 580–587 (1999). 10026102 10.1095/biolreprod60.3.580

[b28] Takahashi T. . Generalized lymphoproliferative disease in mice, caused by a point mutation in the Fas ligand. Cell 76, 969–976 (1994). 7511063 10.1016/0092-8674(94)90375-1

[b29] Manosalva I. & González A. Aging changes the chromatin configuration and histone methylation of mouse oocytes at germinal vesicle stage. Theriogenology 74, 1539–1547 (2010). 20728928 10.1016/j.theriogenology.2010.06.024

[b30] Ju J. C., Tsay C. & Ruan C. W. Alterations and reversibility in the chromatin, cytoskeleton and development of pig oocytes treated with roscovitine. Mol Reprod Dev 64, 482–491 (2003). 12589660 10.1002/mrd.10234

[b31] Manosalva I. & González A. Aging alters histone H4 acetylation and CDC2 A in mouse germinal vesicle stage oocytes. Biol Reprod 81, 1164–1171 (2009). 19641176 10.1095/biolreprod.109.078386

[b32] Zhu C. C. . Effect of mycotoxin-containing diets on epigenetic modifications of mouse oocytes by fluorescence microscopy analysis. Microsc Microanal 9, 1–9 (2014). 10.1017/S1431927614000919 24810297

[b33] Trapphoff T., Heiligentag M., El Hajj N., Haaf T. & Eichenlaub-Ritter U. Chronic exposure to a low concentration of bisphenol A during follicle culture affects the epigenetic status of germinal vesicles and metaphase II oocytes. Fertil Steril 100, 1758–1767 (2013). 24034936 10.1016/j.fertnstert.2013.08.021

[b34] Prigent C. & Dimitrov S. Phosphorylation of serine 10 in histone H3, what for? J Cell Sci 116 (Pt 18), 3677–3685 (2003). 12917355 10.1242/jcs.00735

[b35] Aroor A. R., Restrepo R. J., Kharbanda K. K. & Shukla S. D. Epigenetic histone modifications in a clinically relevant rat model of chronic ethanol-binge-mediated liver injury. Hepatol Int 8 Suppl 2, 421–430 (2014). 26201320 10.1007/s12072-014-9546-4

[b36] Han Z. B. . Interactive effects of granulosa cell apoptosis, follicle size, cumulus-oocyte complex morphology, and cumulus expansion on the developmental competence of goat oocytes: a study using the well-in-drop culture system. Reproduction 132, 749–758 (2006). 17071776 10.1530/REP-06-0055

[b37] Feng W. G. . Effects of follicular atresia and size on the developmental competence of bovine oocytes: a study using the well-in-drop culture system. Theriogenology 67, 1339–1350 (2007). 17420040 10.1016/j.theriogenology.2007.01.017

[b38] Dharma S. J., Kelkar R. L. & Nandedkar T. D. Fas and Fas ligand protein and mRNA in normal and atretic mouse ovarian follicles. Reproduction 126, 783–789 (2003). 14748697 10.1530/rep.0.1260783

[b39] Li H. J. . FasL-induced apoptosis in bovine oocytes via the Bax signal. Theriogenology 80, 248–255 (2013). 23755802 10.1016/j.theriogenology.2013.04.002

