# Supplementary material for: The relationship between apoptosis, chromatin configuration, histone modification and competence of oocytes: A study using the mouse ovary-holding stress model
Source: Sci Rep. 2016 Jun 20;6:28347. doi: 10.1038/srep28347 (PMC4913248; doi:10.1038/srep28347)
Supplement: Supplementary Information [file srep28347-s1.doc]

Supplementary information

**Title: The relationship between apoptosis, chromatin configuration, histone modification and competence of oocytes: A study using the mouse ovary-holding stress model**

Running title: Oocyte apoptosis and chromatin configuration

Juan Lin^1^, Fei Chen^2^, Ming-Ju Sun^2^, Jiang Zhu^2^, You-Wei Li^2^, Liu-Zhu Pan^1^, Jie Zhang^1^, and Jing-He Tan^1,2,3^

1.College of Animal Science and Veterinary Medicine, Shandong Agricultural University, Tai-an City 271018, P. R. China

2.College of Life Science, Northeast Agricultural University, Harbin, 150030, P. R. China

3. Corresponding author: Jing-He Tan, College of Animal Science and Veterinary Medicine, Shandong Agricultural University, Tai-an City 271018, Shandong Province, P R China; Phone: 0538-8249616; FAX: 0538-8241419; Email: [tanjh@sdau.edu.cn](mailto:tanjh@sdau.edu.cn)

Supplementary figure and table legends

Supplementary Fig. S1. Live imaging of chromatin configuration changes in prepubertal mouse oocytes during culture at 39°C. Phase contrast and fluorescence images were taken at 15-min intervals, and the chromatin was pseudo-colored red. While Panel A shows an oocyte undergoing a NSN to c-NSN transition, Panel B shows an oocyte transforming from IN to c-IN configuration. Original magnification ×400.


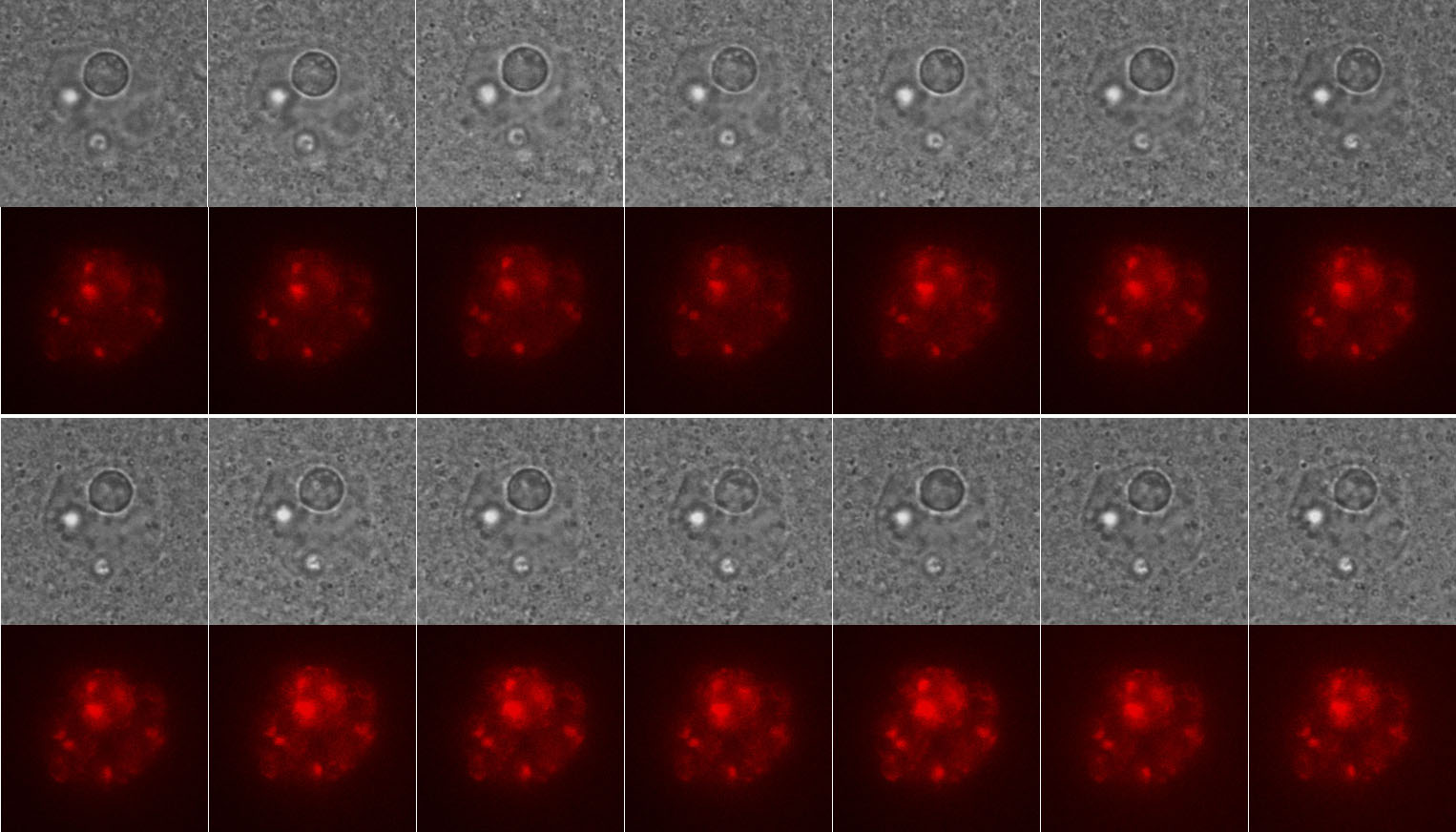


**Phase contrast**

**Fluorescence**

0 min

60 min

120 min

180 min

**A**


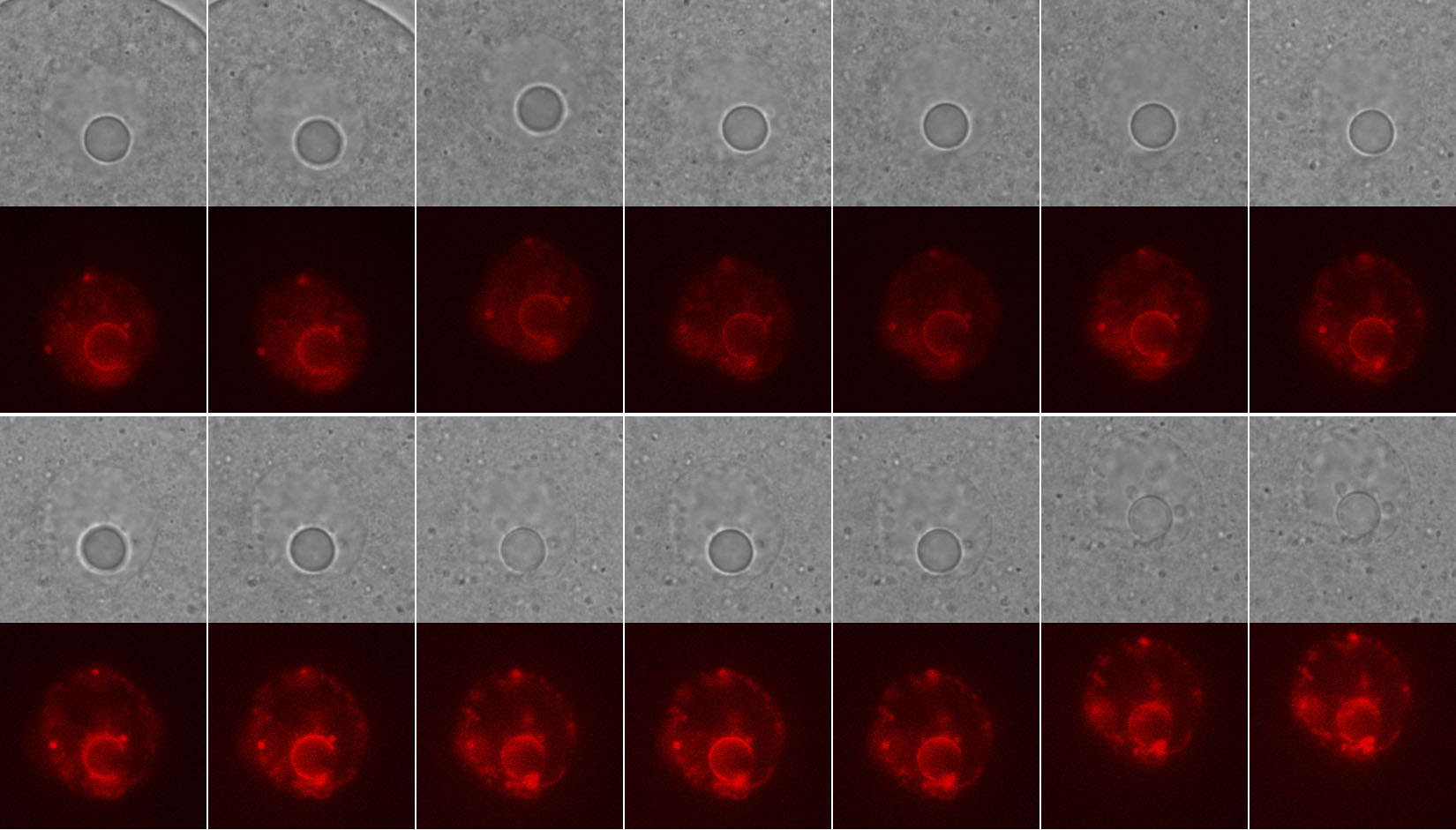


**Phase contrast**

**Fluorescence**

0 min

60 min

120 min

180 min

**B**

Supplementary Fig. S1. Live imaging of chromatin configuration changes in prepubertal mouse oocytes during culture at 39°C. Phase contrast and fluorescence images were taken at 15-min intervals, and the chromatin was pseudo-colored red. While Panel A shows an oocyte undergoing a NSN to c-NSN transition, Panel B shows an oocyte transforming from IN to c-IN configuration. Original magnification ×400.
